# Exploring implementation outcomes in the clinical trial context: a qualitative study of physician trial stakeholders

**DOI:** 10.1186/s13063-023-07304-1

**Published:** 2023-04-27

**Authors:** Kristian D. Stensland, Anne E. Sales, Varsha K. Vedapudi, Laura J. Damschroder, Ted A. Skolarus

**Affiliations:** 1 Department of Urology, Dow Division of Health Services Research, University of Michigan, NCRC, Building 16, 100S-12, Ann Arbor, MI 48109 USA; 2 Department of Learning Health Sciences, University of Michigan, Ann Arbor, MI USA; 3 University of Missouri and Department of Family and Community Medicine, Sinclair School of Nursing, University of Missouri School of Medicine, Columbia, USA; 4 Center for Clinical Management Research, VA Ann Arbor Healthcare System, Ann Arbor, MI USA; 5 University of Michigan, Ann Arbor, MI USA

**Keywords:** Clinical trial improvement, Implementation science, Qualitative research, Outcomes frameworks

## Abstract

**Introduction:**

Cancer clinical trials can be considered evidence-based interventions with substantial benefits, but suffer from poor implementation leading to low enrollment and frequent failure. Applying implementation science approaches such as outcomes frameworks to the trial context could aid in contextualizing and evaluating trial improvement strategies. However, the acceptability and appropriateness of these adapted outcomes to trial stakeholders are unclear. For these reasons, we interviewed cancer clinical trial physician stakeholders to explore how they perceive and address clinical trial implementation outcomes.

**Methods:**

We purposively selected 15 cancer clinical trial physician stakeholders from our institution representing different specialties, trial roles, and trial sponsor types. We performed semi-structured interviews to explore a previous adaptation of Proctor’s Implementation Outcomes Framework to the clinical trial context. Emergent themes from each outcome were developed.

**Results:**

The implementation outcomes were well understood and applicable (i.e., appropriate and acceptable) to clinical trial stakeholders. We describe cancer clinical trial physician stakeholder understanding of these outcomes and current application of these concepts. Trial feasibility and implementation cost were felt to be most critical to trial design and implementation. Trial penetration was most difficult to measure, primarily due to eligible patient identification. In general, we found that formal methods for trial improvement and trial implementation evaluation were poorly developed. Cancer clinical trial physician stakeholders referred to some design and implementation techniques used to improve trials, but these were infrequently formally evaluated or theory-based.

**Conclusion:**

Implementation outcomes adapted to the trial context were acceptable and appropriate to cancer clinical trial physician stakeholders. Use of these outcomes could facilitate the evaluation and design of clinical trial improvement interventions. Additionally, these outcomes highlight potential areas for the development of new tools, for example informatics solutions, to improve the evaluation and implementation of clinical trials.

## Background

Cancer clinical trials aim to advance science, ensure standard of care through protocolization and are considered by many to be the best management for patients with cancer [1]. In these ways, a clinical trial itself can be considered an evidence-based practice. However, cancer clinical trials often fail to meet enrollment goals, prespecified endpoints, and timelines [2, 3]. Taken together, clinical trials can be considered complex, evidence-based interventions with substantial benefits for patients and society, yet suffering from poor implementation [4].

Prior attempts to improve trial implementation have had limited success, due at least in part to a lack of defined frameworks for trial design, evaluation, and improvement [5]. In addition to an enlarging body of clinical trials literature, there have been calls for prioritizing a focus on clinical trial improvement and analysis [6]. Applying implementation science approaches to the clinical trial context could help structure contextual assessments, define implementation outcomes, and inform intervention design to improve trial implementation and success. For example, we previously adapted Proctor’s Implementation Outcomes Framework to the trial context to address these knowledge gaps and target trial improvement and evaluation strategies [7, 8].

In brief, implementation outcomes are a measure of implementation success and exist as both an intermediate precursor to the success of a given practice and as a target for improvement. In other settings, implementation outcomes (e.g., *adoption*, *penetration*, *feasibility*) can measure why evidence-based interventions are not reaching anticipated levels of effectiveness. For example, a smoking cessation program may not be effective in lowering smoking rates in the real world because not many centers are actually using it, i.e., *adoption* of the program is low. Investigating reasons for the low *adoption* through a determinants framework such as the Consolidated Framework for Implementation Research (CFIR) can then identify context-specific targets for implementation strategies and interventions to overcome the identified barriers [9].

We have suggested applying a similar approach to the clinical trial context [8]. For example, answering a trial question requires enrolling sufficient participants. Problems with low enrollment may be due to low *adoption* of the trial by providers (i.e., physicians are not offering enrollment in the trial) or low *penetration* (i.e., a low proportion of eligible patients are enrolling in the trial). Each of these implementation outcomes represents a different issue likely to respond to a different improvement intervention. For example, a service to identify trial-eligible patients (e.g., an informatics solution to “flag” eligible patients in the electronic medical record during clinic visits) could help improve *penetration* of a trial to eligible patients. The same intervention may not be effective if physicians are not offering a trial at all (i.e., trial *adoption* is low). In these ways, specifying the exact outcomes of interest serve as both a measure of trial implementation and a target for trial improvement.

However, the extent to which this approach aligns with current trial practices, and the acceptability and appropriateness of these concepts to real-world trial stakeholders, needs to be better understood prior to further development and application. As clinical trials are complex multilevel interventions with numerous invested parties, the implementation approach could focus on many targets and include many stakeholder groups, all with potentially differing goals, barriers, and facilitators of trial design and enrollment behavior. Specifying exact targets and contexts for different groups, and identifying where these determinants overlap and diverge, will be critical to design and tailor trial improvements. To begin this process, we focused primarily on clinical trial enrollment as the targeted evidence-based practice to be implemented, and limited our initial interviewee group to cancer clinical trial physician stakeholders for multiple reasons.

Our evidence-based practice specification was based on our prior work identifying poor enrollment as the most common reason for trial failure [3]. While there are other factors limiting optimal impact of trials, low enrollment seems to be the reverse salient preventing trial progress. Additionally, enrollment on a trial, per se, can be considered the standard of care for management of cancer, fitting as an evidence-based practice needing improvements in implementation. For most patient-facing trials (e.g., interventional cancer trials), this decision to enroll in a trial is reliant on the patient-physician decision making dyad. We focused on the physician side of this dyad for our present work for multiple reasons. A patient must of course be willing to enroll in and consent to a trial, but the treating physician must either offer the trial or assent to enrollment. Indeed, prior work has suggested that most cancer patients will enroll on a trial if offered enrollment, and to an extent has explored patient-perspective determinants of trial enrollment [10]. We posit the physician side of trial enrollment (i.e., considering trials and offering enrollment) as the rate limiting factor in trial enrollment. In other words, it seems based on prior work and perspectives from patients that the primary problem with trial enrollment may be on the side of the physician, not the patient. Moreover, in addition to offering enrollment on trials, physicians also design trials, serve on institutional review boards, data safety monitoring committees, protocol review committees, and administrative boards overseeing trial design and conduct. In these ways, within the larger group of “physicians” there are numerous stakeholder roles with potentially different perspectives and incentives related to trial design and conduct. These represent roles that are not generally held by trial participants, and thus represent a distinct perspective from participants. This also addresses one of the “Top 10” prioritized research questions from the clinical trials PRioRiTy study: “what are the barriers and enablers for clinicians/healthcare professionals in helping conduct randomized trials?” [6] For these reasons, while future work will incorporate perspectives from other trial stakeholders, we began our investigation by interviewing cancer clinical trial physician stakeholders.

Taken together, clinical trials are critically important and can be considered evidence-based practices with poor implementation. We proposed the use of implementation science frameworks in the clinical trial context, but how components of these frameworks could be understood or applied in trials in the real world is unknown. For these reasons, we studied the consideration and use of cancer clinical trial implementation outcomes, adapted from Proctor’s outcomes, through semi-structured interviews with cancer clinical trial physician stakeholders.

## Methods

As shown in Table 1, we previously adapted Proctor’s Outcomes Framework to the trial context [7, 8]. To explore how each of these outcomes was considered and assessed by cancer clinical trial physician stakeholders in trial design, conduct, and/or regulatory management, we designed a semi-structured interview guide (Supplementary Materials). We piloted this interview guide via mock interviews with two members of our investigative team prior to launching our cancer clinical trial physician stakeholder interviews. We used each of the adapted outcomes in a prompt to assess how each of these were considered and measured by cancer clinical trial physician stakeholders. We in general took an approach of asking for each of a given trial implementation outcome, how the outcome was considered and approached by cancer clinical trial physician stakeholders, and how important the outcome was considered for the overall success of a trial. For example, we previously gave an example of trial *feasibility* as the degree to which it is possible to meet trial enrollment goals. During our interviews, we asked interviewees: “I’m interested in your thoughts on trial feasibility. How have you assessed the *feasibility* of your trial reaching its goals?” We then expanded on these thoughts, for example, “how do you assess how feasible it is to meet enrollment goals in the anticipated timeline of the trial?” and “how do you consider eligibility criteria with respect to the feasibility of a trial enrolling?” Similarly, we evaluated outcomes such as *sustainability* by asking “how much do you consider *sustained* ability to enroll once a trial is opened?” We additionally tailored our interview guide to explore specific physician trial stakeholder roles, when appropriate. For example, a physician member of the Clinical Trials Support Unit was asked about their own experience and approach to the trial outcomes, and then asked how the Clinical Trials Support Unit as a body would approach these outcomes, and to what degree these outcomes influence action by the group.

**Table 1:** Implementation outcomes framework applied to the clinical trial context

Proctor’s implementation outcome	Example in the clinical trial context
Acceptability	Perceived existence of equipoise between intervention arms
	Anticipated or possible benefit over existing options
	Acceptable anticipated side effect profile
	Reasonable participant logistics (e.g., number of clinic visits, distance traveled to trial site)
	Reasonable additional clinical burden (e.g., minimal additional biopsies or other invasive procedures)
Adoption	Proportion of providers offering clinical trials to patients
Appropriateness	Question is amenable to a clinical trial
	Trial design is appropriate for trial question
Feasibility	Possible to meet enrollment goals
	Timeline for enrollment and completion is reasonable
	Anticipated effect size is reasonable
Fidelity	Amount of intervention group crossover
	Adherence to trial protocol including follow-up
Implementation cost	Cost of trial administration
	Cost of trial intervention vs. standard of care (during trial)
	Cost of additional trial staff required
	Cost of additional study components (surveys, labs, scans, biopsies)
Penetration	Proportion of eligible patients being offered trial
	Proportion of eligible patients offered trial who enroll on the trial
	Proportion of patients in global population represented by trial eligibility criteria
Sustainability	Maintenance of accrual rates after trial opens
	Sustained physician interest (i.e., physicians continue offering trial to patients throughout trial period)
	Continued provision of standard of care after trial concludes

*From Stensland *et al*., Applying Implementation Frameworks to the Clinical Trial Context *[7]

Next, we purposively selected 15 cancer clinical trial physician stakeholders from our institution for interviews, representing multiple cancer subspecialties (urology, genitourinary medical oncology, radiation oncology, gynecologic oncology, hematologic oncology, breast medical oncology), trial-related roles (principal investigator, institutional review board, data safety monitoring board, protocol review committee, departmental clinical research team, cancer center leadership, clinical trial support unit leadership), and trial sponsor types (institutional/intramural, NIH/cooperative group, philanthropic organization, industry). All interviews were then conducted by a single interviewer (KDS) via the Zoom videoconferencing platform between July and September 2021 and were roughly 45 min in duration each. Verbal consent was obtained prior to interviews. Interviews were recorded and transcribed, then manually corrected by two coders (KDS, VV). Transcripts were imported into NVivo version 12 (QSR International, released March 2020). Each transcript was individually coded by two authors (KDS, VV) and reviewed together, with discrepancies resolved by consensus. Emergent themes from each outcome were developed collectively and representative quotes for each theme selected (Table 2). During interview coding and theme development, it was felt by the investigators that no new themes or significant ideas emerged with additional interviews, and we agreed thematic and data saturation for this population was reached. This study was considered exempt from full IRB review as human subject research with minimal risk by the University of Michigan Institutional Review Board (HUM#00,198,397).

**Table 2:** Themes from cancer clinical trial physician stakeholder interviews

Outcome	Theme	Representative quotes
Feasibility	Enrollment as key factor in trial feasibility and success	----- “So recruitment is clearly [a barrier]. Are you putting on the patients that will complete the accrual in the timeframe you originally set out? Because if you can’t, then that’s the easiest way for a trial to fail.” [Interviewee 10] ----- “Once we started doing it, we realized that we had fewer patients than we originally expected that fit the clinical context.” [Interviewee 10] ----- “If you’re not seeing these patients now, it’s hard to imagine that some advertisement of the trial is going to make them suddenly show up now.” [Interviewee 1]
	No defined methods for feasibility assessments	----- “Historically it hasn’t been the most scientific of approaches.” [Interviewee 1] ----- “It’s tough as a [trial] reviewer because if you’re reviewing [a trial in] a disease that you do not treat, your understanding of how many patients fit that criteria is unknown, you have no idea. So you’re relying on the study team understanding their disease better than you do, which is typically true and all you can do is say like ‘are you sure you’re going to get this many?’” [Interviewee 10] ----- “One of the groups I work with is very, very diligent looking at metrics, looking at referral populations, looking at sequencing to try and very tactfully choose what trials we’d be able to enroll to. And you try to take yourself out of it, because as physicians our gestalt clinically, as well especially in research, is always wrong: we always think we’re going to win, and it’s just we’re flawed based on that.” [Interviewee 14] ----- “I can’t think of any [feasibility] tools per se. That’s why we have the research team, we have the experience because we have several trials open, so we have that kind of historical view of how well we do as a group, and that certainly kind of gives us the information.” [Interviewee 7]
	Competing trials	----- “Whether it’s going to be feasible to accrue: sometimes you can tell by what other things are going to be competing with that trial with respect to the population, perhaps, that is being targeted to for it to enroll.” [Interviewee 2] ----- “We tend to not have competing trials, there kind of tends to not be a lot of overlap and so that allows you to be pretty consistent.” [Interviewee 4] ----- “We identify competing clinical trials and if they exist, [we have] a very clear prioritization of which studies, what hierarchy, all that would be utilized, that we’re not compromising the conduct of the study.” [Interviewee 5] ----- “I can think of one situation where there’s 5 studies open that are completely overlapping, with the rationale that the force for subsequent studies is we’re going to catch people who weren’t qualifying for the first—and that simply isn’t happening. I think that’s one of the first barriers to success, and probably the most pertinent one to all physicians.” [Interviewee 14]
	Back of the envelope calculations	----- “How many of those types of patients do you see on average per month or per year? And what is the window you’d be accruing in, you know, do you have enough to meet the sample size?” [Interviewee 2] ----- “Essentially our volume of patients is large, so that I was like ‘okay, we can probably get people.’” [Interviewee 3] ----- “It’s much more physician or group impression, and that’s led to significant issues over the years, and so that’s why I think that trying to get some metrics at least to make discussion is important.” [Interviewee 14] ----- “The other is discussing with colleagues: how many of these patients do you see? Which is a very hand wavy and typically over-estimating, I’m learning.” [Interviewee 10]
	Eligibility criteria	----- “Your first question has to be looking at the inclusion exclusion of the trial, your referral base over the last year or how many patients you’ve seen, or actually been referred for investigation of the trial.” [Interviewee 14] ----- “Sometimes you can tell by who’s eligible whether it’s going to be feasible to accrue.” [Interviewee 2]
	Logistics	----- “We do review the trials for feasibility, so for the ability to support the conduct of the study as outlined in the study calendar. Do we have the staff available, is it going to be outpatient, is it going to be inpatient, what kind of additional issues are there for time? Are facilities open?” [Interviewee 12] ----- “There may be some issues we need to look at- so if it’s a study that requires pK blood draws every 2 h for 24 h, it’s just definitely not feasible.” [Interviewee 12] ----- “That [can limit] the potential feasibility, because of the volume or whatever other liquid practical factor in terms of faculty turnover, or availability of all disciplines and necessary things.” [Interviewee 5]
	Low disease prevalence	----- “I think entering into that is the relatively rarity or frequency of the tumor… even if I get a notice of low accrual, I’m sure it’s because it’s a rare tumor, and I will fight to keep it open.” [Interviewee 15] ----- “They’re looking at only about 10% of the patients you approach will have a positive ctDNA, so they’re looking at a pretty big sample size of about 1500 or something.” [Interviewee 6] ----- “When you’re looking for a very narrow molecular marker that may be present in 20% or 10% of patients, it becomes very difficult to recruit to those trials, just because there’s so much screening that goes into it.” [Interviewee 8]
Fidelity	Deviations can affect interpretation of results	----- “Sometimes it will become apparent that maybe the deviations from the protocol are such that it would lead to the data really not being as useful, and the results being potentially undermined a lot, such that the study might not be able to answer this question. So it’s kind of a safety and academic integrity issue.” [Interviewee 2] ----- “The wrong dose of let’s say medicine can be a safety issue, but it also can be an efficacy issue. That may just be a one off, which is unlikely to affect the results too much… [if it’s a] systematic issue that keeps happening… so what’s actually being done compared to what you set out to do, answer the scientific question, and you need to retrain the study team or otherwise enforce it.” [Interviewee 2]
	Importance of trial management group to monitor fidelity	----- “A lot of that is done through both the clinical trial support unit and then also the individual clinic trial coordinators.” [Interviewee 4] ----- “[Our trial coordinator] does a very good job coordinating scheduling, and that sort of speaks to the need for a really good infrastructure—you have to have somebody that’s able to get those things scheduled, to take it off your plate as a provider and make sure things just run smoothly.” [Interviewee 13] ----- “We lean on our trial coordinators in that regard, because they’re doing most of our scheduling and lab ordering for folks on trial.” [Interviewee 15]
	Fewer issues with protocol adherence	----- “That’s been less of a challenge, I would say in my experience, than the pre-screening and identifying patients who could be consented and capturing those at the appropriate time.” [Interviewee 8] ----- “It just gets put in place, and they get their appropriate follow up, and we’ve had patients on study for years or in follow-up for years that rarely we get deviations on, because they get scheduled appropriately.” [Interviewee 13]
Acceptability (Provider)	Perceived equipoise	----- “Sometimes there’s more than one standard of care in a clinical timeframe. And if one is felt to be far inferior to the others, and that is required on the study, that would be something that providers would not see as an acceptable study design, and may not choose to do with them.” [Interviewee 10] ----- “Some of that hesitation is based off of biases of current practice patterns and worrying about how they would change.” [Interviewee 1] ----- “You have to spend a long time thinking about this patient, whether I would be willing to not give him radiation. I decided I wasn’t willing to consider no radiation, so I didn’t offer him the trial, but I think that impacts a lot of trials.” [Interviewee 8]
	Financial incentive	----- “It is no surprise to me that the settings where those trials get done most successfully have different economic incentives than the United States.” [Interviewee 1]
	Provider/investigator disengagement and burnout	----- “It basically all falls on the PI- so I don’t want to be a PI anymore.” [Interviewee 3] ----- “The path of least resistance for me is to just not put people on clinical trials. I think unfortunately what’s actually happening is people are becoming disengaged.” [Interviewee 3]
	Multidisciplinary buy-in	----- “Is there buy-in from collaborators? So oftentimes you’re working with … certain surgical colleagues, medical oncology colleagues, maybe radiologists etc. So is the question interesting to them? Or it could also be their patients on the trial, you know, is that something they’re comfortable with?” [Interviewee 2] ----- “[The trial] should be discussed in a multi-d type setting. Could be at a tumor board, a dedicated meeting, just to make sure everybody’s able to voice any concerns, whether they’re interested in it.” [Interviewee 2] ----- “The first thing is always getting feedback from everybody involved… making sure you have buy-in from everybody.” [Interviewee 5]
	Logistical burden	----- “If it’s just going to add a bunch of tedium, and not offer anything to the patient, that’s not gonna be very palatable for a clinician.” [Interviewee 2] ----- “I see the very strict enrollment criteria and things like trying to get CT scans done in a timely manner, and I know the patient lives six hours away, and I’m going to have a five day window around Christmas to do a cystoscopy on them—and I know there’s not enough support to actually achieve that, and so I don’t want to be a part of this, because then it means filling out amendments, why are we not on time answering questions, overbooking patients on days that I’m not there to try to get a [procedure] in within study window, and it becomes incredibly onerous.” [Interviewee 3] ----- “Things you can do: one is to try and limit the burden associated with accruing people. It’s really easy to start adding on all these correlatives, and quality of life forms, and all of these other things that you want to look at, but it just ends up being more work for both the patient and the physician.” [Interviewee 4] ----- “You’re much less likely to have someone at another institution advocate for your study if it’s hard to do.” [Interviewee 10] ----- “A lot of that has to do with how often they have to see us in clinic… if they need a weekly physician clinic visit that’s a big barrier.” [Interviewee 13]
	Early toxicity/efficacy indicators	----- “Being aware of how people are doing who have been on the trial- if they’re not doing well, obviously that’s going to make a difference with respect to your threshold for putting additional people on the trial.” [Interviewee 2] ----- “We had [a trial] that we stopped putting people on because were uncomfortable with how they were doing once it started to become apparent they were not healing very well, and finally we formally closed it.” [Interviewee 2]
	Direct contact with investigators/providers	----- “[We] discuss with the primary providers and the investigators to get feedback on the scientific premise: if it seems reasonable, if it seems safe, if it’s something that we need to offer their patients, and what they think about the patient population they see.” [Interviewee 5] ----- “We discussed it with the investigators at each site as well as here with the other med oncs to say is this still acceptable given the [other trial] data.” [Interviewee 8] ----- “If there’s not 100% excitement over [a study], then we don’t open it.” [Interviewee 13]
	Patient advocacy	----- “When you’re trying to convince other providers to participate in something, they’re parroting a lot of what patients would tell them in the past.” [Interviewee 10] ----- “We try and design the schedule, let’s say frequency of visits, in a way so it’s going to be more palatable to patients, especially if they’re coming from a distance, fewer visits.” [Interviewee 15]
	“Skin in the game”	----- “You involve people from that site in the design, even in the operations of the trial, so they have some skin in the game and it’s not just you pushing your agenda on them. Even nationally that’s the best way to do it.” [Interviewee 14] ----- “Investigator initiated trials always accrue better, and it’s because somebody’s got skin in the game. And so they’re out there trying to champion at recruiting people there, sending emails to get other people to recruit people.” [Interviewee 4]
Appropriateness	Poor design	----- “Failure would be, not necessarily a negative result, but if it can’t answer the question. So if it’s set up in such a way that it’s intrinsically confounded.” [Interviewee 2] ----- “Are things going to be contaminated pretty heavily by this treatment, so that it becomes harder to interpret something like survival later?” [Interviewee 2] ----- “You need to have an adequately powered trial to answer the question, and if the answer’s no then you’re wasting your time, and the money that comes your way, as well as the patients’ time.” [Interviewee 2]
	Adequate control group	----- “The primary goal has to be that at the end of the trial, the control group, whether that be within the study or historical control, is an accurate representation of that phase of the disease, and that your intervention for the interventional arm was done in a way that you feel at the end there was benefit or not benefit within the statistical framework that you phrased the question. Because of either the design in the patient population or in the statistical design you don’t feel that you actually answered a question that is medically relevant [then a trial failed].” [Interviewee 10]
Acceptability—patient	Expected individual benefit	----- “There are people who are willing to take significant risk so that science can move forward and people 20 or 10 years from now may benefit, but there’s a lot more people who are willing to enroll in a trial if they’re going to get some personal benefit, or at least have a chance of it. And particularly if they have less risk they’re bearing.” [Interviewee 2] ----- “Late phase trials are the most acceptable. There’s this sort of sense when you’re on a clinical trial that you’re getting experimental treatment or placebo or whatever and so I think the acceptance of late phase trials that are randomized is much, much higher.” [Interviewee 13] ----- “Are patients interested at all? If there’s already a 99% cure rate… we’re adding on a toxic therapy, are they really going to be interested to do that?” [Interviewee 14]
	Access to experimental treatment	----- “Ideally, we want a trial that offers something to the patient that they can’t get off trial that will be appealing to them, preferably with some sound reason to believe it may work- you know, Phase 2 data, etc.” [Interviewee 2] ----- “Maybe we use 2:1 randomization to make it a little more palatable, you know a little more likely for patients to want to be in it if it’s 2:1 for new intervention versus standard.” [Interviewee 2]
	Participant logistics	----- “Like travel. Let’s say the study makes people drive to the cancer center three times a week. We have a lot of patients who get three weeks into it and say ‘you know what, I’m done, I don’t want to drive here anymore’ such that we don’t actually get the disease related endpoint.” [Interviewee 10] ----- “Trying to make participation as easy as possible for visit frequency… if a virtual visit would likely be fine you could make that an option” [Interviewee 10] ----- “The more visits or the longer, the less likely people are going to be adherent or compliant” [Interviewee 13] ----- “We always prefer an oral agent [to IV] because obviously more convenience.” [Interviewee 15] ----- “We try to be accommodating in terms of can they get labs for monitoring closer to home? So sparing them that trip, so we try to incorporate those kinds of things to make it more convenient for patients.” [Interviewee 15]
	Minimizing risk	----- “Not letting some of the scientific aspects that you’d like to do get in the way of what needs to be done. We would love to have biopsies before, immediately after, in three months- it’s just that is not at a patient level, for the majority of studies, appropriate.” [Interviewee 10] ----- “We have not had to pull or get this information directly to the patients … just because most of the interventions have been investigated enough that they’re not considered to be too high risk.” [Interviewee 5]
	Reimbursement for visits	----- “I would be honest, it hasn’t been the biggest part patients have talked about, saying ‘oh yeah I got 20 bucks today.’ They just don’t tend to get too excited about it, so I don’t think that changes a lot of their choice to be involved.” [Interviewee 10] ----- “We probably could have paid people a lot more so that was not a huge incentive.” [Interviewee 3]
	Involving patients in design	----- “There was a trial recently where the providers thought there would be no buy-in… so I just approached a couple patients that would have fit the eligibility criteria in clinics and said: hypothetically what would you do here? And they were actually interested in that, and gave useful feedback about moving that forward rather than just submissions off gut reflex.” [Interviewee 14]
Adoption	Directly contacting investigators/providers	----- “You need to have a relationship with the people there. I think just offering it to a center that you don’t know, you’re just going to set yourself up for failure.” [Interviewee 4] ----- “I think it’s very helpful to have someone that you have a personal relationship with that when they’re not accruing you can call them and say hey what’s going on? You agreed to this, do we need to change something?” [Interviewee 4] ----- “I think it’s just having buy-in from the players who are actually going to be the people accruing and having commitments from them that they are engaged, and going to be active and accruing to the trial, or at least attempting to.” [Interviewee 4] ----- “When we’re sitting in the same room with one of [the physicians] I say, this is what trial is going on.” [Interviewee 6]
	Support staff are integral	----- “If resources are available, I really do think that having research coordinators, nurse navigators, people that may even go beyond on helping getting consent and making sure this patient is eligible for the trial [helps with success].” [Interviewee 1] ----- “Supporting portfolio managers so sort of the managerial person to help them run the meetings and the portfolios, lets you not miss anyone.” [Interviewee 11] ----- “We provide staff that can help relieve the faculty of some of the burden. Not all of it, but some of the burden of enrolling patients in clinical trials.” [Interviewee 12]
	Institutional investment	----- “Our chair recognizes that it’s hard to do clinical trial enrollment, but part of our annual review process is that [they] ask how many patients we put on a clinical trial, and I think that just making you say it out loud, is a way to get at ‘are you keeping up the trial that’s been open for seven years?’” [Interviewee 1] ----- “I think it’s really culture…. Supporting the leadership of the teams with salary support… up the game, pushing the aspect for the clinical research leaders.” [Interviewee 11]
	Individual provider engagement	----- “I think one of the major hindrances to accruing people is just a lack of physician engagement.” [Interviewee 4] ----- “I think it’s just having buy-in from the players who are actually going to be the people accruing, and having commitments from them that they are engaged and going to be active and accruing to the trial, or at least attempting to.” [Interviewee 4]
	Difficult to scale trials	----- “I think it’s harder because there’s no way to engage the entire team easily from multiple sites.” [Interviewee 4] ----- “It’s much harder with a large group, exponentially larger and harder- three [providers] is harder than two, and four is much harder than three.” [Interviewee 1]
	Loyalty to existing relationships	----- “Having a strong team, when someone knows ‘ok, this is Kristian’s trial, I’m going to make sure that I help him accrue to this” and I don’t think there’s that same sense when it’s a [multicenter] corporate sponsored trial.” [Interviewee 4] ----- “Making sure the other providers know that the study is important to your career development is another way to try to make sure people are engaged in it.” [Interviewee 10]
	Advertising to physicians	----- “Raising awareness at the weekly provider meeting, GU research meeting, is the way we primarily did it. Using electronic resources like the CTSO website.” [Interviewee 8] ----- “Externally, we try to have websites that are easy to use for external referring physicians to just kind of see what the portfolio of trials is.” [Interviewee 11] ----- “Tumor board helps with that. It’s just a lot of reinforcement. So every tumor board when we discuss patients the first and foremost thing is are they eligible for study?” [Interviewee 13]
	Trial support units/meetings	----- “We have a weekly research meeting that we review the trial, so that’s how we keep track of everyone on trial.” [Interviewee 7] ----- “We provide clinic coordinators, which are either research nurses or non-nurse group research coordinators in the clinic to help… relieve the faculty of some of the burden, not all of it, but some of the burden of enrolling patients in clinical trials.” [Interviewee 12]
	Individual beliefs in trials	----- “You find some people are more aggressive in enrolling and some people aren’t. Some people implement the eligibility criteria in a relatively broad way, and some people there’s a clinical judgment and some are just inherently a little more conservative, they don’t like pushing the envelope so much.” [Interviewee 9] ----- “Some faculty are invested a lot more than others, particularly when it’s not your research and you’re just enrolling patients into somebody else’s trial.” [Interviewee 12] ----- “You can give them information to try to make them feel passionate about your trial, think you can fix everything, but at the end of the day that style of presenting is strongly physician dependent. I think that’s a learned behavior over time, to some degree.” [Interviewee 14]
	Strong PI interest	----- “I also offer to just see and consent and treat all patients if there’s any grumbling about it because I find that’s the fastest, like the path of least resistance, to get people to enroll.” [Interviewee 13]
Implementation cost	Opportunity cost	----- “The other thing is like what the opportunity cost is obviously. You can do it, but then you can’t do a bunch of other potentially more useful things.” [Interviewee 2] ----- “[We are] trying to match our limited personnel and financial resources, as an institution with the larger goals of our whole research group to make sure that we're not doing things that have opportunity costs that could impact our ability to do other things.” [Interviewee 10]
	Lower priority trials not given resources	----- “To some degree, I think the like smaller things like this just get kind of kicked to the side, and you know it's really it becomes a kind of passion project or it dies.” [Interviewee 3] ----- “We have a registry trial that I'm running that has less funds, and that was a bigger struggle for sure, because there wasn't as much funds. And it was not clear if the CTO was going to help or that we could afford it. Those types of trials on a shoestring budget are much harder to arrange and to get going.” [Interviewee 8]
	Cost influences trial design	----- “[Cost] impacts a lot. I mean, I think it kind of goes back to every kind of questionnaire that you want adds cost to the trial because someone has to go through and enter that into the database. And so, big things are just the number of follow up points, so if you wanted to, say, have follow up every month for five years, that would be an absurdly expensive trial just because of the amount of data being collected. So I think you have to be very cognizant about having clinically appropriate follow up, but not more than what is clinically appropriate, and then also really being cognizant of how long you're going to run the trial for because going from three to five years of follow up makes a huge difference cost wise.” [Interviewee 4] ----- “If you’re running a trial, And you know you only have X number of dollars available. you might not do all of the things you would have liked to have done, because you don't have the money, and so you make a trial leaner in order to meet financial goals.” [Interviewee 11] ----- “I think you have to keep things more as simple as possible right? You don't get as many things like additional biopsies and additional correlative studies if you don't have adequate funds for it. Which is too bad because if you're going to go through the effort of getting somebody on trial and doing all the work, you should learn as much as you can about it. But the reality is, that sometimes there’s not money to do those things.” [Interviewee 8] ----- “[Resources] aren’t unlimited right you've got to have enough money cobbled together to run the trial so yeah you might alter your design make it smaller not make it randomized do a number of things because that's just the money that you had to deal with.” [Interviewee 11] ----- “[Cost] is the one of the first things you consider so it's always better to have your trial finished faster and get the corollaries. The faster you run it the more sites you need, more than sites you need, the more expensive it is, so it's always a game of can you run this correctly with your site, or can you afford you know, several other sites and playing the numbers back and forth. Sometimes that means that the trial isn't possible because either you don't have the funding to make it at other centers, you know have buy in or something like that so it's always on your mind.” [Interviewee 14]
	Cost most important but least discussed consideration	----- “Cost, just across the board thinking as globally, bringing those concepts to fruition and completion—the biggest barrier there is cost. But cost is just not even on the radar and never discussed.” [Interviewee 15]
Penetration—eligible population	Reminder in note templates	----- “We at least tried to implement this where you have this smart phrase within MiChart where it says clinical trial eligible true false, and I’m not aware of us using that particularly [to assess penetration] in a comprehensive way.” [Interviewee 1] ----- “We have an infrastructure for evaluating numbers of disease patients specific to certain sites etc. We integrate it into a template… a smart phrase that tells us if patients are clinical trial eligible or not.” [Interviewee 5]
	Peer pressure	----- “We know how many patients [colleague 1] accrued to those trials, I know how many patients [colleague 2] accrued to those trials, and that they were both actively accruing to both of those. So some additional confidence comes with that.” [Interviewee 4]
	Identifying eligible patients is primary issue with trial enrollment	----- “Probably the main reason we don’t accrue as many people is because people just aren’t cognizant enough of this patient is eligible for this trial.” [Interviewee 4] ----- “Getting an email that says you’re seeing this patient on Friday at two, they’re eligible for these two trials [might help with enrollment]. Just having that kind of burnt into your mind going into the consult. My bias is that would be the single easiest way to improve physician engagement.” [Interviewee 4] ----- “If somebody could ever find the eligible patients we’d want that, before we saw them in clinic. The problem is that’s all buried in.pdfs and Epic, so I mean, that would [help improve enrollment].” [Interviewee 6]
	Review of patients at department/multidisciplinary meetings	----- “Many of [the patients] are discussed in the context of multidisciplinary boards of directors, for a team-based approach, to understand those who are trial eligible” [Interviewee 5] ----- “We have weekly research meetings that we review the trial, so that’s how we keep track of everyone on trial. We put potentially eligible, or future eligible patients on this sheet, and those enrolled on a list. It’s trial specific- so if we think of anyone who could be eligible, maybe in the future, even if not right now but potentially in the future, we put their name on the sheet, so that’s really helpful.” [Interviewee 7] ----- “All of the new patients seen that past week are briefly reviewed at a research meeting, in case someone missed one. So occasionally you’ll go oh I didn’t think about X trial, let’s go reach out to them now.” [Interviewee 11]
	Staff identifying eligible patients	----- “We also have our study coordinators screen all patients.” [Interviewee 5] ----- “Having a coordinator who is very assertive about enrolling patients or screening charts and things like that has been helpful.” [Interviewee 13] ----- “If I have a patient that needs a new treatment, I’ll go through the list of trials and see if anything’s appropriate, but it’s more incumbent upon me as a physician and an investigator. We don’t have a very robust pre-screening system.” [Interviewee 8] ----- “My admin basically provides administrative support to the clinical research team, and she pulls from Epic a list of all the new patients seen by the team. Towards the end of the research meeting we run the list, and everyone’s listening to go ‘oh you might have missed this, or did we miss symptom management trials’ or something like that.” [Interviewee 11] ----- “The Clinical Trial Support Unit has been trying to address that. We provide clinic coordinators in clinic to help basically pre-screen patients, so if somebody is coming into clinic that may be a candidate for a clinical trial.” [Interviewee 12]
	Trials as key offer for all patients	----- “I always say trial is an option. I kind of offer to everyone, it’s important for me, so I always mention them to everyone. Even if I think they might not be interested, I can be upfront and mention, as always, this is an option, and I always try to look for one. We may not have anything to offer, then I won’t talk about it.” [Interviewee 7] ----- “There are those patients that you see you’re like oh she’s never gonna go on study but it never hurts to ask.” [Interviewee 13] ----- “If there is clinical equipoise, every patient deserves a clinical trial option.” [Interviewee 9]
	Culture	----- “In our group, I think this sort of first and foremost thing is the culture of not traditionally having trials and so not thinking they’re an important part [of care].” [Interviewee 13] ----- “I think it’s really culture- we’ve developed that culture a long time ago and we’ve been trying to up the game.” [Interviewee 11]
	Individual physician efforts	----- “I see basically all of these patients for the institution; it’s not like I have several members in a group. Some [patients] like to enroll, others don’t, so I don’t have that dynamic—for me if they’re eligible for at rail they’re being presented with clinical trial options, period, guaranteed, across the board. If you think statewide and look at those patients, as a population, that is most definitely not the case.” [Interviewee 15]
Penetration—global	Restrictive eligibility criteria	----- “The narrower the category, the fewer patients you’re going to have- it’s just that simple. You keep adding things you need to have to be eligible or things that they can’t have. The more you add, the smaller the pool’s going to be. It might be appropriate to do so depending on the question, but it’s just kind of a trade-off.” [Interviewee 2] ----- “I think that’s all about making the criteria for entry into the trial broad enough that you can catch patients. That you’re not constantly disqualifying people or that it’s not looking for a needle in a haystack” [Interviewee 8] ----- “If you have a patient population that you think [a trial] would be unsafe, because you think your drug interacts with a drug that you could not take somebody off of, that would be an appropriate exclusion criteria. Outside of that, [eligibility criteria] should be limited, besides also needing some inclusion criteria and exclusion to narrow down the clinical context that you are testing.” [Interviewee 10] ----- “I don’t think it’s for quicker enrollment, I think it’s for offering patients more promising therapies. The quicker enrollment is more PI or institution centered. And really what clinical trials are offering patients treatment options when they are knocked off their other treatment options, and it’s really easy to lose sight of that.” [Interviewee 14]
	Generalizability	----- “We bias towards making trials more reflective of the patients we treat” [Interviewee 1] ----- “That’s not why we do this, at the end of the day. It’s to broaden eligibility criteria. The hope is that we can bring promising therapies to people that are being blocked out because they’re not healthy people, and normally people that are not healthy get cancer at a higher frequency than healthier people. So… I tried to make my eligibility criteria as large as possible” [Interviewee 14]
Sustainability	Maintaining awareness among physicians and trainees	----- “I think it has to do with reminding people that we have this trial; making sure the residents are also aware.” [Interviewee 2] ----- “Our coordinator sends a monthly email of the trials we have open here.” [Interviewee 13] ----- “Raising awareness of [the trial], at the weekly provider meetings, [cancer site] research meeting, is the way that we primarily did it” [Interviewee 8] ----- “At CRT we essentially review the number of patients on every single GU trial … so every week you see how many patients are on each of those trials. … more from the outside, it’s like saying hey remember this just opens this week, these are the patients that are eligible- so really just reminding providers.” [Interviewee 4]
	Monitoring trial enrollment longitudinally	----- “We really scrutinize this on a regular basis in the context of the CRT and make sure that we’re appropriately enrolling.” [Interviewee 5] ----- “A couple of our rare tumor trials have stayed open a really long time, because they’re rare tumors and we put on like one patient every three years. But it’s sort of a continual reminding of people, hey we have this open, and if you have anybody let’s put them on or consider them.” [Interviewee 13]
	Drop-off in trial enthusiasm	----- “When a trial first opens up we do a really good job of getting people on the study and then once you’ve had a couple on and maybe they didn’t do so great, then the enthusiasm goes down and maybe less people are put on.” [Interviewee 8] ----- “We have trials that haven’t had patients on for a while, and you raise awareness every week, but I feel like after the first initial burst of patients it becomes harder to get people engaged in it.” [Interviewee 8] ----- “Some people to start a trial for a reason and then just jump on to something else, or open up a new trial that competes with the existing trial, and then you see another reason why you don’t hit your enrollment mark sometimes. So dropping something in the middle, because you’re excited, you’re distracted by other things that are coming along the way. It’s not the right thing, but it does happen.” [Interviewee 9]
	Anticipating new results	----- “I think the barrier that occurred is the new data that came out… making it harder to enroll, but also maybe squelching some of the signal there.” [Interviewee 8] ----- “You can anticipate it, you can build it in where you say if other drugs are approved in this space, they can also be used in the control arm. Usually if you do have that, the IRB will want you to adjust if one of those things happens with a protocol amendment.” [Interviewee 10] ----- “One thing we did not anticipate that may have slowed us down a little bit, even though we did complete, was shortly after we started [a landmark agent study] was published which meant that was going to be the new standard of care, which we hadn’t built into our study. So there was a question when that data came out: how much would that impact our study? Would we still be able to recruit?” [Interviewee 8] ----- “Sometimes our treatment paradigms change, so you may have designed a study with a certain treatment paradigm, but that treatment paradigm changed by the end.” [Interviewee 10]

## Results

In general, interviewees were excited to discuss potential avenues to improve clinical trials, in particular clinical trial enrollment. The adapted trial outcomes overall were well understood and accepted by cancer clinical trial physician stakeholders. We framed our questions and analysis initially around the conceptualization of each of Proctor’s implementation outcome and then probed early understanding of barriers and facilitators to each outcome. *Feasibility* and *implementation cost* were the most frequently considered outcomes as reported by our interviewees. While *adoption* and *penetration* were important, these were less often considered formally by our interviewees. In general, even when there was awareness and consideration of these outcomes, there were few specific ways to operationalize or measure them within the existing trial infrastructure. We present more specific results grouped by each of the implementation outcomes as follows.

### Feasibility

Feasibility was frequently raised by interviewees, often unprompted, as a key issue facing trial planning. In general, aspects of feasibility most important to interviewees were identifying a sufficiently large potential study participant population and considering eligibility criteria. Interviewees reported enrollment as the key factor in trial feasibility and success, as poor enrollment was reported by an interviewee as “the easiest way for a trial to fail” [Interviewee 10].

Despite this stated importance, few interviewees reported a formal method for assessing eligible populations. This concept was intended as distinct from power analysis (i.e., determining the number of participants and events needed for the desired level of confidence in an anticipated effect size), and instead reflects how many eligible patients are available for a given trial in a given location. When asked how a trialist might estimate the number of patients potentially eligible for a trial, most interviewees report relying on estimates in a way that “historically hasn’t been the most scientific approach” [Interviewee 1]. Interviewees had awareness of how one could estimate the number of eligible patients (i.e., to measure feasibility), but there was no reported concrete pattern or method of how or when these approaches were used, and it was reported that some of these technologies have not been “leveraged to where we need to do trials” [Interviewee 1].

Other important themes related to determinants of feasibility included trial logistics, disease prevalence, and the existence of competing trials. Most interviewees reported trial logistics (e.g., staffing, frequency of lab draws) as important considerations for trial design but these were felt by interviewees to be generally well-handled by current review mechanisms.

Disease prevalence was considered more critical for trial feasibility. For some conditions, an interviewee reported the “volume of patients is so large” [Interviewee 3] a feasibility assessment was essentially unnecessary, whereas it was essential for rare conditions where recruitment is often difficult leading to long trial duration. Similarly, competing trials were reported as having significant influence on trial feasibility and enrollment, endorsed by one interviewee as “one of the first barriers to success, and probably the most pertinent one to all physicians” [Interviewee 14].

### Implementation cost

The cost of running a trial significantly influences trial feasibility and design. Despite cost reported by one interviewee as “one of the first things you [should] consider,” [Interviewee 14], trial costs were felt by others to be underemphasized, and sometimes “not even on the radar and never discussed” [Interviewee 15]. However, trial design itself was reportedly reliant on costs, with “secondary endpoints… highly contingent on available funding” [Interviewee 5]. At a system level, these costs have implications for the approval and funding of other trials. The concept of opportunity cost was used to describe these issues by some interviewees, where funding one trial means “you can’t do a bunch of other potentially more useful things,” [Interviewee 2], including both investigators designing new trials and institutions/sponsors selecting trials for funding.

Aside from selecting correlative studies, other trial design changes were suggested by interviewees as implementation cost-containment measures, including decreasing sample size or follow-up time, omitting randomization, or reducing the number of trial sites.

### Acceptability—provider

Acceptability to providers is a key step in trial enrollment, as these providers are most frequently the gateway for patients to access trials. While interviewees did not report a formal way for measuring acceptability of a trial, the concept was well understood and clearly applicable to trial conduct. We found that a major determinant of provider-level acceptability was the logistical burden of a trial. One interviewee reported that trials can create “more work for both the patient and the physician” [Interviewee 4], sometimes adding “a bunch of tedium” [Interviewee 2] to clinical care. This may be due to more frequent visits or dictated timing of certain studies. Interviewees also reported economic considerations, where lost productivity due to seeing fewer patients, or potentially randomizing patients away from highly reimbursed procedures, has a direct financial impact on providers. Combined, these factors have implications for trial enrollment, with some providers not offering trials, with one interviewee feeling “the path of least resistance for me is to just not put people on clinical trials” [Interviewee 3]. These factors may also lead to less uptake of trials at additional sites and an increased workload on trial principal investigators to make up for shortfalls. This may increase disengagement and provider burnout, with one investigator stating: “it basically all falls on the [trial principal investigator], so I don’t want to be a [trial principal investigator] anymore” [Interviewee 3].

The increased logistical burden on providers suggests a trial must be meaningful to a provider for them to overcome the barriers to participation. As many interviewees reported, this is in part accomplished through direct engagement between investigators and providers to discuss interest in and acceptability of proposed trials, as well as receive input on the design of trials. These methods were also felt by one interviewee to get “skin in the game” [Interviewee 4] from providers to improve adoption and enrollment.

An important component of provider acceptability was the perceived equipoise of a trial. There were strong provider beliefs and “biases of current practice patterns” [Interviewee 1], as put by one interviewee, that can make a trial’s premise less acceptable. For example, interviewees reported that some physicians may not be willing to potentially randomize a patient to not receive a given treatment. One interviewee described a case where “I decided I wasn’t willing to consider no radiation, so I didn’t offer him the trial” [Interviewee 8]. This can also affect trials with multiple treatment modalities that may have similar historical efficacy but vastly different methods of administration, such as radiation therapy versus surgery for prostate cancer.

The acceptability of a trial could also change over time. This presents difficulties in enrolling to trials as evidence evolves, for example, if a new standard of care emerges that was not included in the original trial protocol. Even without external evidence, early indications of toxicity or efficacy while outcomes remain blinded could influence “your threshold for putting additional people on the trial” [Interviewee 2], according to one interviewee. A trial described by an interviewee had decreased enrollment “once it started to become apparent [patients] were not healing very well” [Interviewee 2], leading to closure of the trial. It is important to consider these issues and work in concert with data safety monitoring boards to optimize safe, sustained enrollment to trials.

### Acceptability—patient

There was substantial overlap in aspects of acceptability for providers and patients. Notably, we did not directly interview patients, but patient considerations were a large factor in investigators’ trial decisions. As such, our interviewees’ responses reflect physician’s *perception* of acceptability of trials to patients, and must not be considered to replace acceptability evaluated directly with patients. Many physician interviewees highlighted a patient advocacy component to designing and implementing trials, reportedly looking to “parrot a lot of what patients would tell them” [Interviewee 10]. The logistics of trial participation, such as travel time to trial sites, were seen as major barriers to trial acceptability and enrollment. Investigators described approaches to trial design that could decrease participation burden and increase acceptability, such as minimizing the number of return visits, identifying sites closer to a patients’ home for lab draws, or converting to virtual visits when possible.

Even when facing these burdens, many patients will seek trials to access experimental treatment. This was reported by interviewees to be a major driver for some trials, particularly in early phases, where a “trial that offers something to the patient that they can’t get off trial” [Interviewee 2] can more easily enroll. For other trials, using strategies like a 2:1 randomization scheme (i.e., a higher chance of receiving the experimental therapy) can “make it a little more palatable” [Interviewee 2] for patients to enroll on an experimental trial.

The expected benefit from trial enrollment was also highlighted by interviewees, primarily as a barrier to trial participation where perceived benefit was low. For example, in conditions with “already a 99% cure rate” [Interviewee 14], enrollment on a trial “adding a toxic therapy” [Interviewee 14] would be difficult. Late phase trials were also perceived by interviewees to be more acceptable than earlier trials, perhaps due to perception of receiving a more “proven” active treatment. Other fringe benefits, such as financial incentives, were not felt by interviewees to significantly impact enrollment to trials.

### Adoption

Patient trial participation hinges on provider trial adoption. Interviewees reported mechanisms to identify how many physicians were enrolling patients onto trials, but how to increase this adoption was less clear. A difficulty commonly reported by interviewees was individual provider engagement, i.e., speaking directly with other physicians about the trial. Low levels of enthusiasm for trials in general, or for a specific trial concept, were felt by interviewees to lead to poor trial adoption and enrollment rates. Part of this engagement was an individual’s belief in the importance of trials, with one interviewee reporting “some faculty [are] invested a lot more than others” [Interviewee 12]. However, some engagement may be modifiable, such as through individual, direct communication with providers through existing relationships. Interviewees reported that continued communication with providers allows for investigators to check in on trial progress and address changes, and physical co-location at clinical sites permits in-person reminders of ongoing trials at the time of clinic visits. Furthermore, interviewees reported using advertising at a group level, such as through multidisciplinary tumor boards, departmental meetings, or research meetings as a potential adoption improvement strategy.

It can be difficult to apply these techniques at scale, however. Some approaches work at an individual level, for example, one interviewee reported they individually “just see and consent and treat all patients… I find that’s the path of least resistance to get people to enroll” [Interviewee 13]. However, this approach is likely unsustainable at multiple sites or with higher enrollment goals. Similarly, individual meetings to increase adoption within a group of 2 or 3 providers was feasible, but expanding to larger groups was reported by an interviewee to be “exponentially larger and harder” [Interviewee 1], particularly if multiple sites were included.

Interviewees reported that institutional investment and support staff may help address some of the issues with adoption. Support staff resources were reported by interviewees to aid immensely with recruitment and improve the likelihood of providers adopting trials. Direct investments in resources for trials could support more of these measures. Additionally, indirect investments from an institution, such as trial involvement being considered as part of promotion or reimbursements, may contribute to a culture of inquiry and encourage trial adoption by providers.

### Penetration

While there were methods to assess both provider adoption of trials and how many patients total enroll in a trial, assessing the proportion of eligible patients enrolled in a trial (i.e., penetration) was reported by interviewees to be much more difficult. In part, this assessment has the same root challenge as enrolling patients: identifying who exactly is eligible. Interviewees reported that while the number of patients approached for a trial was typically recorded and easily accessed, the total number of eligible patients presenting to clinic (i.e., the denominator of total eligible patients) was difficult to measure through the electronic medical record. Despite difficulties measuring penetration, there were some attempts to improve trial conduct that target improved penetration.

One method was to manually identify eligible patients. Some interviewees reported using study coordinators or administrative support to screen all new patients for potential trials. Interviewees reported reviewing patients in a multidisciplinary setting within certain groups, such as “in the context of multidisciplinary boards” [Interviewee 5] where new patients are reviewed and eligibility for trials from within the groups’ portfolios could be assessed. Some interviewees also reported multidisciplinary tumor boards as a good opportunity to recommend trial involvement.

Other attempts to improve penetration relied on aspects of culture and peer pressure. Some interviewees emphasized trial involvement as a standard offer to every cancer patient, considering a trial “always an option” [Interviewee 7], to aid in increasing penetration. Highlighting peer trial enrollment performance was also used by some interviewees to increase confidence in enrolling to trials. As with encouraging adoption, including enrollment numbers during performance review and consideration for promotion, at least in academic settings, was also reportedly used to attempt an increase in trial penetration. Interviewees also emphasized the importance of broad eligibility criteria both for enrollment purposes and to ensure representation and access to therapies for as many patients as possible.

### Sustainability

While trials may be successful when first launched, interviewees reported it may be more difficult to sustain this conduct over the trial period. Interviewees reported a drop-off in trial enthusiasm “after the first initial burst of patients” [Interviewee 8]. This could be from newly opened trials competing with the existing trial, providers forgetting about an existing trial, or loss of enthusiasm for a trial as early results are reported. Some strategies reported by interviewees to combat this loss of enthusiasm were reminding providers and trainees about specific trials, sending email reminders of existing trials, and strategies similar to encouraging adoption and penetration (e.g., reminders at tumor boards or research meetings). Another issue raised by interviewees was the emergence of new data or treatments affecting trial equipoise or rationale. Interviewees suggested trials could be designed with potential adaptability in mind, or amended to adjust for these new treatments.

In general, interviewees did not report issues with sustained protocol adherence or follow-up. Interviewees felt well-supported by institutional trial infrastructure and support staff resulting in good participant retention and follow-up on trials.

### Fidelity

Fidelity to trial protocols was not reported as a major issue for cancer trials at our institution. Interviewees did suggest a hypothetical issue with protocol deviations affecting interpretation of trial results, but this was reportedly not often seen in practice. Overall, fidelity, including protocol adherence and follow-up/retention, were reported as “less of a challenge” [Interviewee 8] than other aspects of trial conduct, mostly due to strong support from trial coordinators and support units.

### Appropriateness

We did not explicitly frame an interview question to ask about appropriateness, as asking about trial appropriateness in pilot interviews was off-putting to pilot interviewees, and it was felt that the data gathered from directly asking about appropriateness would most likely only yield comments on improving trial design. Over the course of the interviews, interviewees did comment on the importance of a well-designed trial as paramount to evidence generation. From the perspective of many interviewees, a trial that was not appropriately designed to answer a reasonable question cannot be a successful trial, even if the trial meets its goal enrollment. The design features referenced by interviewees to be important aspects of appropriate trial design included sample size and effect size for power estimates, and the selection of an adequate control arm for randomized trials.

## Discussion

We explored implementation outcomes and early determinants of success in the clinical trial context through semi-structured interviews with cancer clinical trial physician stakeholders at our institution. Our findings highlight important underemphasized components of clinical trial conduct, as well as areas that are largely functioning well from the investigator perspectives. We found implementation outcomes to be well understood by cancer clinical trial physician stakeholders, and reflective of issues faced in trial design and implementation. Taken together, our findings highlight important targets for trial implementation improvement and evaluation research.

The most important outcome considerations for trial conduct were felt to be feasibility and implementation cost. These implementation outcomes were the most easily understood and most frequently considered by cancer clinical trial physician stakeholders. While issues of implementation cost could largely be addressed by increasing funding for trials, a more realistic aim may be improving trial efficiency. Understanding feasibility and its assessment may make trials more efficient, but operationalizing assessments of eligible patients at scale is a complex undertaking. Perhaps for this reason, despite endorsing the importance of feasibility, investigators described few formal methods of trial feasibility assessment. Development of these methods, and testing their use and effects on trial enrollment and success, is an important area for future trial implementation work. This may be of particular use in determining additional site placement in multisite trials, or in identifying locations for trials with industry, government, or cooperative group sponsors who are institution-agnostic with respect to trial sites.

Other aspects of feasibility assessment are labor-intensive, and thus costly, and may be amenable to informatics solutions [11]. Identification of patients eligible for clinical trials is a major challenge, likely increases cost of trials, and also impacts the evaluation of trial penetration to eligible patients [12]. While approaches to patient identification such as through natural language processing could help identify patients, these innovations must also be tested within trials to evaluate their impact on enrollment [11].

These improvements to assessing feasibility could result in more efficient and more cost-effective trials. This may help address a critical problem, as the cost of running trials was highlighted by multiple interviewees as a major barrier to implementing trials. Cost can potentially limit trial design elements such as collecting correlative endpoints or the duration of follow-up, discourage the launching of new trials, or create incentives to study only certain types of interventions in trials. Cost may also be particularly important in certain contexts where funds are limited and design features may be directed more strongly by sponsors. While targeting improvements in specific trial outcomes such as adoption and penetration have value per se, improving the efficiency of trial conduct and specifically trial enrollment has the potential to decrease trial cost, removing a barrier to success and facilitating more and better clinical trials. It will be important when designing trial improvement interventions to consider the cost of these interventions relative to the benefit to trials to maximize their use and encourage adoption by trialists and trial sponsors.

Conceptualizing trial enrollment as affected by adoption (i.e., uptake by providers) and penetration (i.e., proportion of eligible patients enrolling on a trial) could be helpful for targeting trial improvement interventions. Investigators in our study had strategies implicitly aimed at these components, but investigators in general were not explicit about these targets. Certain strategies, such as advertising at tumor boards, could impact both adoption and penetration, but such strategies may not work in all contexts. Prior work examining multidisciplinary meetings has highlighted the promise of improved recruitment, but also challenges with team dynamics affecting trial enrollment [13, 14]. Despite understanding these concepts and applying informal strategies (e.g., speaking directly with colleagues to increase trial adoption, advertising trial to attempt to increase penetration), there was little formal assessment of exactly how many physicians were enrolling patients (i.e., adoption) or an exact evaluation of penetration (i.e., how many patients were enrolling relative to the eligible local population). Similarly to assessments of feasibility, there was an interest in understanding penetration, but very little formal assessment or logistical capacity for its evaluation. There is a clear need for future work in this space, both to improve trial conduct and to measure the success of enrollment improvement interventions.

Overall, these concepts were easily understood and seemed acceptable to investigators, suggesting future trial improvement strategies using these terms could be an effective way to efficiently measure and improve trials. Use of this standardized language can also facilitate adaptation of implementation strategies developed in other complex intervention contexts to clinical trials. Our approach is complementary to efforts to assess trial conduct using behavioral theories, such as qualitative work aiming to improve recruitment to trials, by framing trial improvement within an implementation science model to facilitate the development and targeting of specific interventions [4, 15, 16]. Similarly, our work could add to efforts, such as those from the QuinteT group, to improve enrollment through qualitative work [17]. Indeed, prior work applying qualitative methods to efforts at recruitment has highlighted similar themes to those found in our work, especially difficulties in identifying eligible patients [18]. Our work can add to these findings by applying an implementation lens to the identified barriers, adding an interventional implementation component to the qualitative work.

An initial application of these measures is in the evaluation and improvement of ongoing trials. For example, use of our outcomes framework approach allows for endpoint measurement in trial improvement evaluations, termed studies within a trial (SWATs) [19]. For example, investigators mentioned email reminders to providers about ongoing trials to improve enrollment. A hypothetical trial improvement study, or SWAT, could randomize a set of trials to email reminders or no email reminders, and measure how many providers offer the trial (adoption) and the proportion of eligible patients enrolled (penetration). This would improve upon prior endpoints of simply “enrollment” or “success.” Such studies present opportunities to evaluate the effectiveness of informatics solutions to support trial implementation, such as algorithms to identify trial-eligible patients, best practice advisory “pop-up” alerts in the electronic medical record, or automated email audit and feedback on trial enrollment performance.

In addition to ongoing trials, our results also emphasize the importance of initial trial design. While many of the strategies used by investigators and suggested by our frameworks look to improve existing trials, it is critical to evaluate the appropriateness and feasibility of clinical trials prior to implementing them. Our interviewees emphasized that a trial must be worth doing (i.e., a trial must be appropriate for the question asked). Part of developing this question may be incorporating physician and patient input to optimize acceptability to both patients and physicians prior to beginning the trial. Despite the stated importance of trials being acceptable, our interviewees did not express a formal method of determining acceptability of trials to physicians or patients. This is another area in need of exploration to improve trial design. Ideally, we can decrease waste by improving trial design initially, and identifying and de-implementing trials doomed to fail before they begin or when they have become unsustainable.

While our interviewees reported few issues with fidelity to trial protocols or follow-up initially or sustained over the trial period, this may reflect our strong institutional trial infrastructure. Other institutions without substantial clinical trial support units may struggle more with protocol adherence or sustained follow-up. These differences may also explain the infrastructural or “trial effect” explaining part of the patient benefit of trial enrollment [20, 21]. Additionally, we focused our investigations on cancer trials, predominantly reflecting interventional trials. Some issues with trials of other types (e.g., trials of complex interventions such as smoking cessation programs) might face more barriers to fidelity and sustainability. Future work is planned to investigate these outcomes and determinants in different local contexts and for other intervention types.

Our initial experience exploring implementation outcomes in the trial context with cancer clinical trial physician stakeholders at our institution was generally positive, though our study does have limitations. The first limitation of this study was the narrow scope of participants; we only interviewed one type of clinical trial stakeholder, physicians. Many other disciplines and types of stakeholders are involved in clinical trials and will be incorporated in future studies. However, we did include interviewees from multiple cancer specialties and trial roles. Interviewing only physicians also limits understanding of the patient perspective, particularly for considerations of trial acceptability to patients. Understanding the physician perspective alone can inform trial considerations, and future work will compare physician and patient perspectives on trial design and conduct. Additionally, all interview subjects are members of our own institution, limiting the potential transferability of these perspectives to other contexts, particularly trialists at community sites. Future studies are needed to assess responses in other contexts.

Our initial qualitative exploration of clinical trial implementation outcomes identified targeted areas for trial improvement and supports the acceptability and appropriateness of implementation outcomes in the trial context. Use of the adapted implementation outcomes framework was well understood by cancer clinical trial physician stakeholders, aligned with their understanding of trial processes and barriers, and highlighted nuanced outcomes that could enhance trial improvement and measurement strategies. Applying these outcomes highlighted determinants worthy of further exploration, and future directions for trial improvement research through implementation science methods.

## Conclusions

Through semi-structured interviews with cancer clinical trial physician stakeholders, we explored implementation outcomes in the clinical trial context and found targeted areas for future clinical trial improvement and evaluation strategies.

## Data Availability

The datasets generated during and/or analyzed during the current study are not publicly available as they are direct transcripts of human subject interviews, but are available from the corresponding author on reasonable request.
